# A Qualitative Study on Surgeon Perceptions of Risk Calculators in Emergency General Surgery

**DOI:** 10.1097/AS9.0000000000000567

**Published:** 2025-04-21

**Authors:** Claire B. Rosen, Amanda L. Bader, Sanford E. Roberts, Justin T. Clapp, Scott D. Halpern, Margaret L. Schwarze, Rachel R. Kelz

**Affiliations:** From the * Department of Surgery, Hospital of the University of Pennsylvania, Philadelphia, PA; † Department of Anesthesiology & Critical Care, Hospital of the University of Pennsylvania; Philadelphia, PA; ‡ Department of Medicine, Hospital of the University of Pennsylvania; Philadelphia, PA; § Department of Surgery, University of Wisconsin School of Medicine and Public Health; Madison, WI.

**Keywords:** clinical decision-making, evidence-based medicine, informed consent, qualitative research, risk calculators, shared decision-making, surgeon

## Abstract

**Objective:**

To understand how surgeons perceive risk calculators in emergency general surgery (EGS).

**Background:**

EGS involves complex decision-making of operative and nonoperative management. Risk calculators can aid in shared decision-making and informed consent.

**Methods:**

We performed semi-structured interviews among emergency surgeons within 1 healthcare system to explore perceptions of risk calculators in EGS. Interviews were completed until thematic saturation, transcribed, coded in duplicate, and analyzed using inductive analysis within a modified grounded theory framework to generate theory regarding surgeon perceptions of risk calculators in EGS.

**Results:**

Among 20 interviewees, the mean age was 45. We identified dominant themes related to the concerns of and benefits of using risk calculators within EGS, both in situations of clear and unclear best treatment options. Surgeons questioned risk calculator validity and noted that a lack of health numeracy can limit their use. Risk calculators were seen as helpful for communication, consensus building, informed consent, and litigation mitigation. The ideal risk calculator should have low activation energy for use, incorporate relevant data and accurate prognostication, and provide actionable, easily interpretable output. Education for providers and patients on risk calculator availability and use is necessary.

**Conclusions:**

Although surgeons may initially question the data produced by risk calculators in EGS, they identify several potential virtues to their bedside use when optimal treatment options are and are not clear. The ideal risk calculator for use in EGS should be convenient and relevant. Future studies are needed to explore patient perceptions and to directly observe patterns of risk calculator use.

## INTRODUCTION

Respect for patient autonomy has been the leading bioethical principle in modern medical practice in the United States over the past few decades.^1–3^ As such, there has been a major focus on patient-centered care and shared decision-making (SDM), in which physicians and patients collectively work to enact medically sound care plans in line with patient values.^1,3,4^ Emergency general surgery (EGS), which represents 7% of all hospitalizations annually in the United States (nearly 3 billion admissions per year),^5^ is a heterogenous field encompassing both operative and nonoperative management, allowing for multiple treatment options, often for medically complex patients.^6–9^ For optimal SDM in EGS, the surgeon must be able to evaluate available medical evidence, to communicate the evidence in support of or against both operative and nonoperative treatment options, and consider and discuss how those options fit with the patient’s life goals, values, and preferences. To support this process, there has been a proliferation of decision-making tools, such as risk calculators.^10–13^

Risk calculators are often designed to incorporate patient-specific factors to generate personalized risk prediction that can aid in patient care, SDM, and the informed consent process. Unfortunately, many patients are unsatisfied with the amount and type of information shared by providers before surgery,^14–16^ particularly with regard to the quality of life that can be expected after an intervention.^17^ Risk calculators can provide personalized information in a digestible format and can enhance patients’ participation in SDM and the informed-consent process.^15,18^ Although there are many risk calculators for use in EGS that can provide useful and accurate risk prediction for patients (eg, the American College of Surgeons National Surgical Quality Improvement Program [ACS NSQIP] surgical risk calculator, the Emergency General Surgery Frailty Index, the Multimorbid Patient Identifier App, and the Surgical Risk Preoperative Assessment System, among others), they are often underutilized.^13,15,18–20^ A better understanding of how physicians perceive the use of technology-enabled tools, such as risk calculators, in EGS decision-making would be helpful to improve their utility within clinical practice.

As such, this qualitative investigation aims to understand how surgeons perceive the utility of risk calculators in the EGS setting. Rooted in the ideal of SDM, and guided by desires from the interviewed surgeons, we further aim to generate a theory regarding the ideal future state of a risk calculator for use in EGS. This will hopefully serve to improve future risk calculator creation, implementation, and education regarding their utility and application in SDM for EGS.

## MATERIALS AND METHODS

This is a qualitative investigation of surgeons within a single health system, using semi-structured interviews and inductive analysis within a grounded theory framework, to understand and develop theory around how surgeons perceive the use of risk calculators in EGS. The Institutional Review Board at the University of Pennsylvania deemed the study protocol exempt (#851118). Surgeons provided verbal consent for participation. We prepared this manuscript in compliance with the Consolidated Criteria for Reporting Qualitative Studies (COREQ) reporting guidelines for qualitative studies (see eSection1, Supplementary Digital Appendix, for COREQ checklist http://links.lww.com/AOSO/A488).^21^

### Research Team and Reflexivity

We developed the interview guide as an iterative process by study team members, including senior team members with robust experience in performing and teaching qualitative research (J.T.C., M.L.S., and R.R.K.). A single team member (C.B.R.), a postdoctoral research fellow in medical ethics and general surgery resident physician, conducted all interviews, with another team member (A.L.B.) observing some interviews for quality control. All participants knew the interviewer before the interview, and many knew of their established outcomes research in EGS. We informed participants that the study goal was to “understand how surgeons make decisions, specifically in the emergency setting” (see eSection2, Supplementary Digital Appendix, for interview preamble http://links.lww.com/AOSO/A488). We were concerned that revealing our primary goal to be understanding surgeon perceptions of risk calculators in EGS may bias surgeons to answer questions with a greater focus on risk calculators than they would normally have in a clinical scenario, so we did not make specific reference to risk calculators at the outset of the interview. This allowed us to gather information regarding surgeon perceptions of decision-making in EGS, including the role of risk calculators, in an unprompted (and unbiased) way, and with a later greater insight into their perceptions of risk calculators through targeted questioning.

### Participants and Setting

Study participants were practicing surgeons who take emergency surgical calls. We selected these surgeons given the variety of operative and nonoperative treatment options in the emergency setting, along with the availability of a variety of risk calculators used in this setting.^10,13,22^ We used convenience sampling within a single academic medical system which includes a hub tertiary care hospital and multiple smaller academic and community hospitals. We used purposive sampling in selecting surgeons from a variety of surgical specialties evaluating patients with EGS pathology (trauma, critical care and emergency surgery, gastrointestinal surgery, and colon and rectal surgery) and with variable years of experience (<1 year, 1–5 years, 6–15 years, >15 years). We selected participants to reflect the gender identification and racial background makeup of the surgical workforce of the institution. We identified possible participants and approached them via email for participation. Of all approaches (n = 26), 2 surgeons (8%) did not respond to the email invitation, 4 (15%) had to cancel due to clinical and personal responsibilities, and 20 interviews (80%) were completed.

### Data Collection

We developed the interview guide based on the research team’s expertise and literature review. There were over 30 revisions of the interview guide, initially developed based on prior work by senior team member (R.R.K.),^23–25^ adapted for the current research study by primary investigator (C.B.R.), and iteratively revised by study team members (R.R.K, J.T.C., M.L.S., S.D.H., S.E.R.) to generate focus and clarity. We pilot-tested the initial interview guide with the first 2 interviews. The semi-structured interview guide consists of open-ended questions and associated prompts, focusing on decision-making when there are multiple viable treatment options and surgeon perception of population-level data and risk calculators (see eSection2, Supplementary Digital Content 1, for interview guide http://links.lww.com/AOSO/A488).

We performed all interviews from a private home office or a private workplace office using Microsoft Teams (Microsoft Corporation, version 1.6.00.11156) between March 2022 and May 2023. We audio-recorded and auto-transcribed interviews through Microsoft Teams, with auto-transcriptions subsequently checked for coherence and edited to exactly match the audio recording. We recorded field notes only when body gestures signified meaning to the words and added these to transcriptions. We deleted all audio recordings after the edited transcription was finalized and saved all transcriptions without names or identifying information. Interviews ranged from 30 to 45 minutes. We did not perform repeat interviews for any participants and participants did not re-check transcripts.

### Data Analysis

Study team members (C.B.R., A.L.B., J.T.C., M.L.S., and R.R.K.) developed and iteratively revised the codebook, beginning after the second interview. The codebook reached stability by interview 10 (see eSection3/eTable1, Supplementary Digital Content 1, for codebook http://links.lww.com/AOSO/A488). Two research team members (C.B.R. and A.L.B.) coded the data using NVivo for Mac (QSR International Inc., Version 14, Release 1.7.1). We held inter-rater reliability meetings after coding every 2 to 3 interviews, with discussion and resolution of any major disagreement. We derived themes from the data via an inductive analysis approach with a constant comparison between codes, concepts, and themes.^26^ Although we expected to reach thematic saturation by 20 interviews, we ensured that thematic saturation was reached by comparing thematic abstraction after coding 14 interviews to a repeat analysis after the completion of 20 interviews. No new themes or conceptual categories emerged in this interval, which suggests that thematic saturation was reached by interview 14. However, we included all collected data in this analysis. We provided participants with quotations to be used in the manuscript and major themes/findings before submission for publication. All participants approved the included data.

## RESULTS

Of 26 surgeons invited to participate, 20 (80%) completed a semi-structured interview. A small majority of participating surgeons specialized in trauma, critical care, and emergency surgery (n = 11, 55%), with others in gastrointestinal surgery (n = 7, 35%), and colon and rectal surgery (n = 2, 10%). Years in practice were relatively evenly spread, and 6 surgeons (30%) had trained at the study institution. Most surgeons practiced at a hub tertiary care center (n = 12, 60%). See Table 1. There were neither overarching differences in the responses between specialty, years of experience, gender, race/ethnicity, nor practice environment noted in a comparative analysis.

**TABLE 1.** Participant Characteristics

Surgeon Characteristics	Total
n (%)	20 (100.00)
Age, mean (range)	44.9 (35, 62)
Female (yes), n (%)	6 (30%)
Race	
White, n(%)	14 (70%)
Black, n (%)	2 (10%)
Asian, n (%)	4 (20%)
Specialty	
Trauma, critical care, emergency surgery, n (%)	11 (55%)
Gastrointestinal surgery, n (%)	7 (35%)
Colon & rectal surgery, n (%)	2 (10%)
Time in current role	
<1 year, n (%)	5 (25%)
1–5 years, n (%)	6 (30%)
6–15 years, n (%)	6 (30%)
>15 years, n (%)	3 (15%)
Trained at current institution (yes), n (%)	6 (30%)
Practice environment	
Tertiary care center (academic)	12 (60%)
Urban community	5 (25%)
Rural community	1 (5%)
Mix of the above	2 (10%)

### Surgeon Concerns About Using Risk Calculators

#### Clinical Scenario

Many surgeons reported that they do not need a risk calculator to inform their decision—that they often can choose a clear best treatment option, especially when the risk of death without surgery is perceived as high (“I don’t think we would have not operated on her because of the risk calculator. You know, like, she still needed surgery.”—interview 3). Surgeons described confidence in their risk estimations based on experiential learning and training, that is, physician heuristics (eg, “I then create my own, kind of, risk algorithm in my mind.”—interview 6). Further, they noted that risk calculators can even cause some element of moral distress, with reports of feeling bad about a high-risk option when it is perceived as the only one available (“If [a risk calculator] tells you that there’s a 30% chance they’re gonna die, […] you don’t feel good.”—interview 12). Regardless of surgeon preferences, surgeons also noted that patients can have established treatment preferences, which can outweigh any calculated risk (eg, “Most people had pretty much made-up their mind”—interview 15) Table 2.

**TABLE 2.** Surgeon Reported Barriers to Using Risk Calculators

**Clinical Scenario in which Best Treatment Option is Clear**
	Physician Perspective	
	Physician heuristics	5: “Every surgeon thinks they know what exactly is the right course […] sometimes wrong, but never in doubt is the surgeon’s creed.” 12: “I didn’t use a risk calculator […] I just knew that it was a high-morbidity operation. And if the risk calculator spit out 30%, what am I gonna do different?”
	Physician moral distress	7: “and you’re like, “great, wish I didn’t know that quite so well.” Then you’re probably not going to use it if there’s not an intervention to help make it better, you just like, ‘well, I’m screwed. I just gotta operate on this old guy.’” 12: “They don’t want to look at [risk estimates] because it can only make you feel worse about what you’re about to do.”
	Patient perspective (physician reported)	
	Patient’s established treatment preferences	15: “I found in many situations [patients] have a clear [opinion]. They’re like, ‘I don’t want surgery, I don’t care what the risks are or how few they are,’ or, ‘I want surgery. I don’t care what the risks, are or what the outcomes are.’ So, you’re in the situation where you’re making the decision about surgery or not, regardless of what the risk calculator tells you.” 4: “Most people had pretty much made-up their mind to proceed [with surgery] and it was putting a number to the high risk, or whatever scary things I was telling them […] but it didn’t like defer anyone or change anyone’s mind from proceeding so far, in my experience.” 12: “The patient perspective almost ends up mattering as much or more than a risk calculator. Like how much of a risk are they willing to take?” B
**Clinical Scenario in which Best Treatment Option is Unclear**	Favored Odds	19: “So there’s that like protective mechanism where you assume, ‘ohh that’s not gonna happen to me, I’ll be in that other percent.’”
	Subjectivity of risk threshold	6: “If I said to a family member that this patient has a 5% chance of living, 95% chance of mortality, I will tell you that the majority of people will take that 5%. And if you offer an operation, or you offer them whatever, on 5%, they’re gonna take it.” 15: “You have a 90% chance of dying. Might be, ‘I’ll roll the dice on what that 10% chance is,’ and, to somebody else, 90% or whatever might be, ‘I’ve had a good life, forget about it.’”
**Health Numeracy**	Physician	8: “[Data] are just statistics […] it’s a matter of not only understanding the data and the evidence and the risks, but also putting them into a context that becomes meaningful as a communication
	Patient	2: “I think people poorly understand 5% chance of something.” 5: “But I don’t think humans, [people], nonmedical people have the capacity in, perhaps the capability to appropriately justify what that means, you know?”
**Characteristics of the Tool**	Internal Validity	
	Face validity	15: “Many people would say even if you click the button that this is an emergency procedure in something like the [American College of Surgeons] risk calculator, [the outcome estimate] might not do justice to the rate of morbidity and mortality associated with the given procedure.” 1: “I guess what I wonder about all scores is like […] are they more accurate than an experienced clinician’s assessment? So like this guy is sicker than he might otherwise appear in the data, but is he sicker than I think he is when I meet him?”
	Content Validity	6: “I don’t use a formal [risk calculator] ‘cause [they] don’t incorporate all of the elements that I may bring it, right? Like, not every patient has cardiac disease completely quantified so that you can fill out a risk calculator.” 15: “In emergency surgery, risk calculators that don’t take into account physiology are tremendously flawed.”
	Research Quality	1: “Any evidence we use contains bias of various kinds.” 6: “Not every study is done perfectly well, for various reasons […] so you end up using lesser studies to try to extrapolate what the right answer is to do.”
	External Validity	
	Generalizability	10: “You also have to look at the populations they were validated in, right? […] if the study population was colorectal surgery, and I jumped to pancreatic surgery and I’m using the [National Surgical Quality Improvement Program] calculator, I don’t know if we’re really comparing apples to apples.” 15: “I find that data is plentiful, but I often find that it doesn’t always fit exactly with the patient I have in front of me who has all these different comorbidities.”
	Impersonal	9: “It’s a little challenging sometimes to apply population-level outcomes to the individual patient […] because the patient’s outcome is either […] zero or one at that level.” 18: “Population-level risk doesn’t apply at the individual level.”
	Convenience	16: “I think it’s time […] [Risk calculators] only take a few minutes, but, in a busy emergency surgery day, that may […] feel prohibitive.” 5: “It seems [whenever] these emergencies happen, you’re on the phone, coming up with a plan with a resident, I never even think to say wait a second, let me put this into my app and see what it says.”

When the best treatment option for a given patient is unclear to the surgeon, surgeons do not universally turn to risk calculators to elucidate clarity. Some patients may use a defense mechanism where they believe unlikely odds to still be in their favor (eg, “like that protective mechanism where you assume, ‘ohh that’s not gonna happen to me”—interview 19), which limits the surgeons’ perceived benefit of using a risk calculator. Furthermore, even “objective” numbers (ie, risk estimates provided from a risk calculator) can be perceived subjectively, and this perception can differ between providers and patients, complicating, rather than aiding, communication (eg, “having a risk of a 10% risk of something, especially like a 10% lifetime risk, is actually pretty high”—interview 19).

#### Health Numeracy

To properly use a risk calculator requires a level of physician health numeracy and statistical literacy. Similarly, there needs to be some level of patient medical literacy and numeracy (eg, “What’s the difference between a 50% risk of a complication and a 75%, or 60%?—interview 16). Without both the ability of physicians to interpret and explain statistics and the patient to understand the statistics and how they apply to their clinical situation, surgeons are concerned that risk calculators can complicate, rather than aid, SDM.

#### Characteristics of the Tool

Regarding the tool itself, surgeons noted concern about the internal and external validity of risk calculators. Regarding internal validity, they wondered whether all risk information for a particular patient is sufficiently included in any risk calculator (eg, “The thing that informed me the most was her [cancer] treatment history, […] I don’t think that there’s a way to incorporate that into the risk calculator.”—interview 16), or if the quality of included research and data is sufficient. Regarding external validity, most interviewed surgeons lamented that risk calculators are developed based on population-level data—and that this data may not be generalizable to the patient or may be too impersonal (eg, “Everybody has an odds of something, or a risk of something, or a percent of something, but it always ends up being 100% for whatever happens to you.”—interview 1). Finally, surgeons reported that, especially in EGS, time is limited, and taking even a few more minutes can prohibit risk calculator use (eg, “It’s just time. If you’re running from clinic to the operating room, back to clinic, to another operating room.”—interview 4).

### Surgeon-Perceived Benefits to Using Risk Calculators

#### Clinical Scenario

When the surgeon noted a clear preferred treatment option, they reported using risk calculators as leverage to convince the patient of their decisions. This takes multiple forms: using data to prove that the risk of surgery is prohibitively high (eg, “If you want to stack the deck against surgery, you can use [risk calculators].”—interview 12), using data to prove that the risk of an adverse outcome is lower with nonoperative management (eg, “So here is a way for me to demonstrate to you that the risk of operating is way higher than the risk of them living with this.”—interview 19), and using data to prove that the risk of surgery is lower than the patient perceives (eg, “ [The risk of surgery] really it’s not as bad as you think.”—interview 1). They also used risk calculators to validate their own heuristically-derived estimations (eg, “[to] prove to myself that this patient had a relatively high mortality no matter what”—interview 14), lending a level of objectivity to their own estimations Table 3.

**Table 3.** Surgeon Reported Facilitators to Using Risk Calculators

**Clinical Scenario in which Best Treatment Option is Clear**	Leverage	11: “When it’s very clear to me, as a clinician, that they cannot survive the operation, but the family or patient say, ‘I want you to do everything you possibly can,’ […] I think that those calculators would be very helpful.” 5: “‘No Sir, you’re 84, you’re on [a blood thinner], you have a reducible inguinal hernia that your family doctor said you needed fixed. But I’m your surgeon. I’m telling you, you don’t need it fixed because […] the risk of strangulation is exceedingly low in your group of patients.’ So, I actually use [risk calculators] to talk a lot of people out of surgery.” 13: “Or the converse - this patient really needs an operation, I know this number is going to show me something that’s gonna help me convince them that they need an operation.”
	Validation	14: “[I used the risk calculator to] prove to myself that this patient had a relatively high mortality, no matter what.”
**Clinical Scenario in which Best Treatment Option is Unclear**	Shared decision making	2: “Because [the risk estimate] frames the conversation about, ‘well, I could not do a surgery here and these are the likely outcomes, or, I could do a surgery and these are the trials and pitfalls that I can anticipate.’” 1: “I like to do things that I think will help, […] and that I think are concordant with [the patient’s] goals and condition. And some of those things you can quantify with a risk calculator.” 10: “It’s certainly most helpful in allowing the patient to determine, or the family to determine, whether surgery is the right thing or not. [The risk calculator] paints a little bit more accurate picture.”
	Complex patient	9: “Doesn’t say operate or not, it just provides a framework for understanding what the outcomes that would, or are expected to be, for the average patient in this condition with these comorbidities. That’s very helpful […] I don’t use that every night but like, maybe once a month, when it’s a really complex patient and a complex decision.” 10: “[Risk calculators] may influence the surgical plan [for example], anastomosis versus colostomy.” 9: “The [American College of Surgeons] risk calculator is especially helpful [for] complex patients, from a medical comorbidity standpoint, [when there are] reasonable alternative [treatment options].”
	Modifiable risk factors	7: “Sometimes it’s about nutrition and, you know, using risk calculators [can inform the need] to get them to a better level of nutrition […] sometimes doing the risk calculator makes you look more carefully at the patient’s labs than you might have done. And so, in those situations, when you discover, like, some sort of awfulness that you’ve managed to just let go by.”
**Communication**	Consensus building	11: “I think it gives me and the patient a place to start from, a common language or perception, ‘cause if I say the risk of this is really high, I may be thinking that’s 10% and they may be thinking that’s 80%.” 14: “One of the downsides of an emergent practice is that you don’t have rapport with most of your patients, at least initially, and so those conversations can be challenging, and having some objective data can be helpful.” 11: “So, if I say, ‘based on this score, your risk of having a complication is 20% and a complication can mean anything from a wound infection to transfusion to return to the operating room to a urinary tract infection, or it could mean death.’ [… the risk calculator] gives us a common place to start.” 3: “The most important role that [the risk calculator] has is it helps people understand how critically ill somebody it is.”
	Informed Consent	13: “When patients ask about, ‘What percent of the time is this going to happen?’ [… the risk calculator provides] specific percentages of the types of times this XYZ would happen.” 14: “Providing some more numbers on what recovery looks like, more than anything else, I think is helpful.” 2: “You can say to them, ‘well, based on thousands of people who have been in the situation that you’ve been in, the likelihood of you having to go to rehab after this is whatever, or the likelihood of you having a complication, and that complication involving going back to the [operating room], is this.’”
	Litigation Mitigation	10: “We pull [the risk calculator] up, do the calculation and we even put it in the epic chart with their with their note, so that it’s recorded that we had that conversation and this is the risk.” 4: “I think in an obtuse way, [the risk calculator] not only like increases the satisfaction with you, but just by being up front, it reduces the risk of them coming back and being unhappy and pursuing some, you know, medical-legal action against a failed surgery.”

When the preferred treatment option was unclear, surgeons perceived that risk calculators may aid in SDM (eg, “Most people haven’t really thought about [what they want in recovery after treatment]. And so providing some more numbers on what recovery looks like, more than anything else, I think is helpful.”—interview 14). They noted the benefit of using risk calculators for medically complex patients (eg, “When you’ve got 18 different comorbidities […] that’s where it’s really so helpful.”—interview 9), especially when there were multiple “viable” treatment options, including assistance in selecting the safest surgical technique (eg, “[calculators] may influence on maybe the surgical plan [like] anastomosis versus colostomy.”—interview 10). Furthermore, surgeons reported that risk calculators can help to detect modifiable patient risk factors that may reduce risk associated with treatment (eg, “You can’t make yourself any younger, but what if your hemoglobin A1C was better?”—interview 7).

#### Communication

Surgeons noted that risk calculators can aid in communication with patients, including building rapport, especially in the emergency setting when there is usually not an already established relationship. Oftentimes, surgeons noted the informed consent process to be a particularly important time to consider the use of risk calculators—to not only provide patients with objective information to help them to understand risks, but to also mitigate potential future litigation (eg, “So that we are going in with our eyes wide open.”—interview 4).

### Comparing Surgeon-Perceived Benefits and Concerns

**FIGURE 1.**
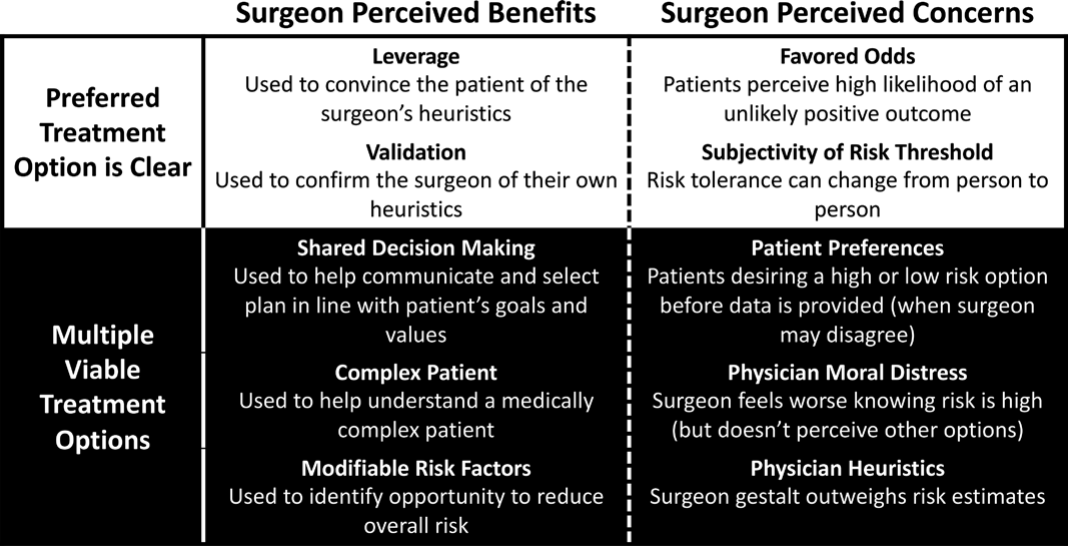
Surgeon-perceived benefits to and concerns about using risk calculators in the emergency general surgery setting in states of a clear preferred treatment option and when there are multiple viable treatment options.

We identified several key points regarding surgeon perceptions (good and bad) of risk calculators EGS, both when there is a preferred treatment option, and when there are multiple viable treatment options, derived from the above analysis and synthesized in Figure 1. These points/themes are fluid, rather than strict, and can be used to help guide both the development of future risk calculators and the education of surgeons and patients in their use. This is particularly important in EGS when there can be a plethora of treatment modalities in use, including operative and non-operative management of patients.

### Future State of the Ideal Risk Calculator

#### Input

Regarding input into an ideal risk calculator, surgeons desired the inclusion of information that they perceive contributes to patients’ risk, like patient physiology and the specific operation. High-fidelity prognostication should be used, along with validated “big data”, which should mitigate some of their concerns regarding the accuracy of risk calculators (Table 4) (as noted in Fig. 1).

**TABLE 4.** Surgeon Desires for the Future State of Risk Calculators for Use in the Emergency Surgery Setting

**Input into Risk Calculator**	MD perception of patient risk	15: “Risk calculators that don’t take into account physiology are tremendously flawed.” 16: “Trying to somehow get more specificity, in terms of what [operation] is gonna be [performed]. I think it can be challenging
	Fidelity	15: “I’m sure with [artificial intelligence] they can continue to throw more and more data into these kind of models, and continue to tweak them and come up with more and more accurate models.”
	Data	10: “A well-established database: it’s validated, it’s used by most institutions, it’s been used for years, so there’s a lot of backing to it.” 2: “Big data is gonna be really useful in being able to prognosticate, based on various different things.”
**Output from Risk Calculator**	Actionable	1: “It might be useful to say […] because of this patient’s specific set of comorbidities, they’re gonna do better with surgery vs better with nonoperative management.” 7: “I think when you have a tool that you feel like will do something for you, then you’ll use it.” 1: “[What I want to know is] whether knowing about those [risks] gives you the opportunity to manage them better and change those outcomes.”
	Patient-Centered	15: “As we get more and more quality of life data, in patient-reported outcomes and things like that, that kind of information might be important: how many or not go back to work, how many are independent, whatever this, and that—it would be great to have […] for an individual patient, what’s important to them.”
	Ease of interpretation	18: “I think for me, I’m fine with percentages and things, but I’m sure that to demonstrate to patients, some sort of graphical visualization would be helpful for patients to understand […] what are they looking at.”
**Logistics**	Integration with EMR	6: “[It has to] live in the [electronic medical record], where it’s already pulling all the data and I don’t have to separately enter it, and [it would be all inclusive so that I don’t] feel like I’m only entering half of the relevant things.”
	Convenience	10: “Make some of these tools a little bit more user friendly, right? I mean, I can ask Alexa, “blah blah blah,” and the Alexa is gonna tell me, you know, the information I need.” 15: “I think you need to be able to literally do it 24 hours a day. You need to probably be able to do it on your phone […] they need to be practical.”
**Education**	Physician	13: “We talk about [risk calculators] in training. I think that training somebody to do it initially […] to use it every time in a clinic setting, or even in the emergency department setting [would be ideal].” 18: “We should teach [the clinical application of statistics] in medical school.”
	Patient	13: “Making patients aware [of risk calculators….] so that [they] could use it, or if they don’t know how to use it, [they could] speak to their doctor about, ‘what’s my risk of XYZ after this operation.’”

#### Output

Regarding output from the risk calculator, many surgeons felt strongly that actionable information is more valuable than simple risk predictions—particularly when there are multiple management options, and a risk calculator can help to identify the option associated with the lowest overall risk for an individual patient. Regarding risks reported, they felt that long-term and patient-centered outcomes, like patient quality of life and ability to work, are important (eg, “Anything that will either prevent the patient from getting to their goal, or will make them worse than they were when they walked in the door.”—interview 20). To aid in communicating the output with patients, there should be consideration as to the visualization of the output—perhaps with a focus on graphical descriptions or information that the average patient could easily interpret (eg, “Color coding […] green, yellow, red. I do think that that’s a visual that’s helpful ‘cause it’s so dumbed down, but also ingrained in your subconscious that those colors are ‘good or bad’, or ‘yes or no’, ‘go or stop’.”—interview 19).

#### Logistics

Many surgeons remarked that they feel as though they should be using risk calculators more often in their practice (eg, “You know, in theory [risk calculators] should be applied universally.”—interview 10). However, many noted that they do not often use them. A risk calculator should be convenient for the surgeon to use, likely requiring low activation energy, and should involve integration with existing medical tools (like the electronic medical record).

#### Education

Finally, there should, of course, be proper training in risk calculator use, interpretation, and communication of results for providers. But there also needs to be education for patients regarding the availability and interpretation of risk calculators.

## DISCUSSION

This qualitative analysis, using interviews of surgeons regarding their perception of risk calculators in EGS, identified several salient themes. When a surgeon identifies a preferred treatment course, surgeon heuristics, surgeon moral distress, and established patient preferences can reduce surgeons’ inclination towards using a risk calculator. However, surgeons see value in using risk calculators as leverage and to validate their heuristically-derived risk estimations. When a surgeon perceives that there are multiple reasonable treatment options, or when the surgeon is on the fence about the least harmful option, the subjectivity of risk thresholds and favored odds can raise concerns about using risk calculators. On the other hand, surgeons perceive that risk calculators assist with consensus building, informed consent, and litigation mitigation. Gaps in physician and patient health numeracy can lead to risk calculators complicating, rather than aiding, communication. Though surgeons voiced some concerns regarding the validity and convenience of existing risk calculators, many reported feeling as though they should use risk calculators more often. Risk calculators should be easy and convenient to use (with integration into existing medical records), should incorporate relevant data and high-fidelity prognostication, and should provide actionable, patient-centered, and easily interpretable output.

When making decisions in EGS, surgeons often rely on intuition and fast thinking, rooted in prior experiences and pattern recognition over facts or data (ie, heuristics).^27–30^ A recent study by Marwaha et al^31^, which compared patient risk prediction via surgeon intuition to that of the ACS NSQIP surgical risk calculator, found preoperative surgeon intuition alone to be an independent predictor of patient outcomes, but that predictions derived from the ACS NSQIP were more robust (and there was no evidence of enhanced prediction when surgeon intuition was combined with ACS NSQIP). Another study, in which surgeons were randomly assigned to receive risk-calculated data along with a clinical vignette (versus clinical vignette alone) to predict surgical risk, found that surgeons provided with risk-calculated data provided more accurate risk assessments with less variation, though there was no difference in the likelihood of recommending surgical management.^32^ As such, the value of heuristics that was reported by many surgeons in our study does not seem like an inappropriate approach to patient assessment. However, physician intuition can run the risk of influence from cognitive bias.^28,31,33^ It is unrealistic and likely impossible to entirely overcome cognitive bias in surgical decision-making. However, it is important for surgeons to be aware of this bias, and many of our participants reported recognition of this phenomenon, an important first step in physician behavior change.^34^ For example, participant 7 reported, “I use [risk calculators] as I please, I think I, yeah, completely nonobjective.” Further, many remarked that they feel as though risk calculators should be used less selectively, or even universally. Similarly, it has been noted in the literature that despite surgeons believing their individual judgment to be the best “evidence” (over textbooks, journals, clinical practice guidelines, etc.), true evidence-based medicine (of which risk calculators are one form) can be useful in even daily decision-making.^35,36^

A more universal application of risk calculators to aid in decision-making and communication could be a step towards mitigation of some cognitive bias, but surgeon behavior change is predicated on having a conducive environment.^36–38^ As such, the activation energy required for a surgeon to use risk calculators should be reduced as much as possible. Risk calculators should be integrated into the existing physician workflow. This could take the form of embedding them into medical records or the integration of mobile apps with existing recorded patient history and data. And, although risk calculators should not be perceived as a sufficient form of evidence to entirely make a decision, they can aid in the SDM process, perhaps even bringing to light patient values and preferences if patient-centered outcomes are included in the output of such tools. Risk calculators can be an adjunct to other published communication tools for improving SDM and the informed-consent process, like the “best case/worst case” communication tool.^39,40^ Importantly, when introduced to risk calculators like the ACS NSQIP, patients report a desire of using them before surgical consent.^41^ Furthermore, the use of a risk calculator need not exclude surgeon gestalt—in fact, the addition of a “Surgeon Adjustment Score” to the ACS NSQIP has even been shown to improve the calibration of risk prediction modeling.^42^ Education on these aspects of risk calculator function would likely improve surgeon interest in and use of such tools.

To develop the ideal risk calculator (to help SDM in EGS), it will be important for this tool to have appeal to both surgeons and to patients (and their family members). To best understand the attributes most important to patients, further investigation is needed, though patients do report interest in using risk calculators.^18,41^ Our study has highlighted some major concerns that surgeons have regarding risk calculators, when the preferred treatment is clear or when there are multiple viable treatment options, including the subjectivity of risk thresholds, preference for their own heuristics, and the moral distress that can go along with the high risk of morbidity or mortality. As such, an ideal risk calculator need not only be a useful, easy-to-interpret, and convenient tool, but the utility of any risk calculator requires appropriate training for surgeons/providers regarding how to best use tools like risk calculators (and evidence-based medicine as a whole in the informed consent process),^43–46,47^ how to best communicate with patients (even with patients who may have low health literacy or numeracy),^47,48^ and how to combat their own moral distress that can go along with treatment options with surgeon-perceived low chance of benefit.^49^

### Limitations

The data used in this analysis was generated from surgeon interviews—neither surgical decision-making in clinical practice nor conversations between the interviewed surgeons and their patients were observed. As such, our results can only discuss what surgeons report to be their perceptions of using risk calculators in EGS, rather than the truth of practice. In discussing their perceptions of risk calculators in EGS, there were instances in which surgeons extrapolated their perceptions to those of risk calculators overall, not just within EGS, which could limit some of our specificity, though it still provides important insight into surgeon perceptions. Furthermore, all participants were from a single tertiary care system, and although this system includes a variety of hospital environments, it is in one geographic, mostly urban, region. All participants, also, had previously met the team member conducting the interviews. Though this offers benefit in that participants were able to speak candidly with a trusted colleague, the prior relationship could introduce bias into the way the interview questions were answered. Finally, this study does not include the perceptions of patients, an important part of SDM.

## CONCLUSIONS

This qualitative study of surgeon-reported use of risk calculators in the EGS setting identified several benefits to using risk calculators in clinical practice, and several situations in which surgeons perceive drawbacks to using them. Although surgeons may initially question the data produced by risk calculators in EGS and fear miscommunication, particularly when there is a lack of health numeracy, they identify several potential virtues to risk calculator bedside use, when preferred treatment options are, or are not, clear. These benefits include communication and enhanced SDM (particularly in the realm of informed consent), validation of surgeon gestalt, litigation mitigation, and identification of modifiable patient risk factors. The ideal risk calculator for use in EGS should be convenient and relevant. As surgeons are only part of the SDM process, and as this study relies on surgeon-reported perception rather than actual use, future investigation regarding patient perception of risk calculators in this setting and direct observation of risk calculators could be valuable next steps in improving overall utility of risk calculators for SDM in EGS.

## ACKNOWLEDGMENTS

C.B.R., A.L.B., S.E.R., J.T.C., S.D.H., M.L.S., and R.R.K.: participated in study conception and design, data analysis and interpretation, and revised the manuscript. C.B.R., A.L.B., and R.R.K.: participated in data acquisition. C.B.R.: drafted the original manuscript. All authors approved the final manuscript for publication.

## Supplementary Material
