# Supplementary material for: A Qualitative Study on Surgeon Perceptions of Risk Calculators in Emergency General Surgery
Source: Ann Surg Open. 2025 Apr 21;6(2):e567. doi: 10.1097/AS9.0000000000000567 (PMC12185096; doi:10.1097/AS9.0000000000000567)
Supplement: Supplementary file 1 [file as9-6-e567-s001.pdf]

Supplementary Digital Appendix

for

A Qualitative Study on Surgeon Perceptions of Risk Calculators in Emergency General Surgery

Claire B. Rosen, MD MSME
Amanda L. Bader, MD
Sanford E. Roberts III, MD MSCE
Justin T. Clapp PhD MPH
Scott D. Halpern, MD PhD MBE
Margaret L. Schwarze MD MPP FACS
Rachel R. Kelz, MD MSCE MBA FACS

From the Department of Surgery, Center for Surgery and Health Economics, The University of Pennsylvania Perelman School of Medicine, Philadelphia, PA (CBR, ALB, SER, RRK), the Department of Anesthesiology & Critical Care, The University of Pennsylvania Perelman School of Medicine (JTC), the Department of Medicine, The University of Pennsylvania Perelman School of Medicine, Philadelphia, PA (SH), the Leonard Davis Institute, Philadelphia, PA (JTC, SDH, RRK), and the Department of Surgery, The University of Wisconsin School of Medicine and Public Health, Madison, WI (MLS).

Table of Contents

**eSection 1: Consolidated Criteria for Reporting Qualitative Studies (COREQ)……………………………**	p.3
COREQ Checklist………………………………………………………………………………………………	p.12
**eSection 2: Interview Guide and Preamble…………………………………...……………………………..**	p.6
Information for Participants/Preamble……………………………………………………………………………	p.6
Semi-Structured Interview Guide………………………………………………………………………………	p.7
**eSection 3: Analysis……………………………………………………………………………………………..**	p.11
eTable1: Codebook……………………………………………………………………………………......	p.11
**References……………………………………………………………………………………………………….**	p.15

eSection 1: Consolidated Criteria for Reporting Qualitative Studies (COREQ)

COREQ Checklist[1]

No	Item	Guide questions/description	Investigation
Domain 1: Research team and reflexivity			
Personal Characteristics			
1.	Interviewer/facilitator	Which author/s conducted the interview or focus group?	C.B.R.
2.	Credentials	What were the researcher's credentials? *E.g. PhD, MD*	M.D., M.S.M.E.
3.	Occupation	What was their occupation at the time of the study?	Post-doctoral research fellow in medical ethics, general surgery resident physician
4.	Gender	Was the researcher male or female?	Female
5.	Experience and training	What experience or training did the researcher have?	with didactic training and experience in qualitative interviews
Relationship with participants			
6.	Relationship established	Was a relationship established prior to study commencement?	Yes
7.	Participant knowledge of the interviewer	What did the participants know about the researcher? e.*g. personal goals, reasons for doing the research*	Personal goals, prior research in emergency general surgery, ethics career
8.	Interviewer characteristics	What characteristics were reported about the interviewer/facilitator? e.g. *Bias, assumptions, reasons and interests in the research topic*	Goals of understanding how surgeons make decisions, knowledge of prior research
Domain 2: study design			
Theoretical framework			
9.	Methodological orientation and Theory	What methodological orientation was stated to underpin the study? *e.g. grounded theory, discourse analysis, ethnography, phenomenology, content analysis*	Grounded theory
Participant selection			

10.	Sampling	How were participants selected? *e.g. purposive, convenience, consecutive, snowball*	Purposive, convenience
11.	Method of approach	How were participants approached? e.*g. face-to-face, telephone, mail, email*	email
12.	Sample size	How many participants were in the study?	20
13.	Non-participation	How many people refused to participate or dropped out? Reasons?	6 (no response, cancellation due to clinical or personal responsibilities)
Setting			
14.	Setting of data collection	Where was the data collected? e.*g. home, clinic, workplace*	Private home office and private workplace office
15.	Presence of non-participants	Was anyone else present besides the participants and researchers?	A.L.B
16.	Description of sample	What are the important characteristics of the sample? *e.g. demographic data, date*	Emergency surgeons, specialty, years in practice, gender, race/ethnicity, training at study institution, practice environment
Data collection			
17.	Interview guide	Were questions, prompts, guides provided by the authors? Was it pilot tested?	Yes pilot tested, interview guide in supplement
18.	Repeat interviews	Were repeat interviews carried out? If yes, how many?	No repeat interviews
19.	Audio/visual recording	Did the research use audio or visual recording to collect the data?	Audio recording
20.	Field notes	Were field notes made during and/or after the interview or focus group?	Field notes of body gestures
21.	Duration	What was the duration of the interviews or focus group?	30 to 45 minutes
22.	Data saturation	Was data saturation discussed?	Yes, thematic abstraction compared after interview 14 to

			after interview 20
23.	Transcripts returned	Were transcripts returned to participants for comment and/or correction?	No, but utilitzed quotes returned for comment and/or correction
Domain 3: analysis and findingsz			
Data analysis			
24.	Number of data coders	How many data coders coded the data?	Two
25.	Description of the coding tree	Did authors provide a description of the coding tree?	Yes, see supplement
26.	Derivation of themes	Were themes identified in advance or derived from the data?	Derived from data
27.	Software	What software, if applicable, was used to manage the data?	NVivo for Mac
28.	Participant checking	Did participants provide feedback on the findings?	Yes
Reporting			
29.	Quotations presented	Were participant quotations presented to illustrate the themes / findings? Was each quotation identified? e*.g. participant number*	Yes
30.	Data and findings consistent	Was there consistency between the data presented and the findings?	Yes
31.	Clarity of major themes	Were major themes clearly presented in the findings?	Yes
32.	Clarity of minor themes	Is there a description of diverse cases or discussion of minor themes?	Yes

eSection 2: Interview Guide and Preamble

Information for Participants/Preamble:

Thank you for taking the time to meet with me today and for agreeing to participate in my study. I am interested in understanding how surgeons make decisions, specifically in the emergency setting. Your participation is completely voluntary – you may stop interviewing at any point or choose not to respond to any questions that you don't feel comfortable answering. Your answers are confidential, and they won't impact your work in any way. There are no right or wrong answers to the questions I'll ask you – I'm interested in hearing your own thoughts, perspectives, and experiences related to this topic. For the purpose of this interview, try to think of me not as a surgical resident, but as someone without surgical training. If I ask you questions to which you'd assume I know the answers, it's to get a more-complete understanding and your words that will really help a lot with our future analysis. I'd like to record our conversation – this is completely voluntary and just to ensure that I capture everything we discuss, while being able to devote my attention to you and our conversation in place of scribbling notes. The recording will be transcribed and deidentified, and your name or any other identifying information will never be associated with anything you say when the findings of this study are disseminated. If you have any questions, please ask me at any time during the interview, or you can contact me afterwards. If this all sounds okay to you, with your permission I would like to start the recording and interview?

Semi-Structured Interview Guide

- When seeing a consult patient in the emergency room, sometimes things are really clear: a patient should or should not get an operation – it can feel like an almost automatic process. I'm interested in understanding how you think through treatment decisions when a patient has an acute or urgent surgical problem that may or may not be best treated with surgery – when there isn't that clear path. Tell me what comes to mind when I bring this up?

    - Are there any types of clinical scenarios that you are thinking about?
    - To you, what is the difference between the easy/automatic scenarios and when you really have to stop and think about what you should do?
        - What types of things make it harder to see a clear path?
            - How do you think about these things?
            - What is most important to you?
            - What types of things don't really matter in these situations?

  [if surgeon seems confused by this scenario, or is not talking about emergency surgery-type equipoise situations, can add…"What about when you're worried that surgery isn't the best idea?"]

- Okay, now I'd like to get a deeper understanding of how you deliberate about what do when there isn't a clear path, despite a surgical diagnosis. I want you to think about a specific patient when the best course of action didn't seem automatically clear – [if applicable] maybe think more about the patient you mentioned with [insert previously mentioned surgical problem] just a few moments ago?

  [pause]

    - Tell me ["tell me more", if already mentioned in brief] about the situation?
    - Walk me through weighing the possible options?
    - Tell me about what influenced your treatment decisions and thought processes?

  [if mention of prior experience/pivotal case]

    - Tell me more about how that experience or case influences how you decide what to do?
        - How do you compare that experience or case with other cases?

  [if mention colleagues, collaboration with colleagues]

    - Tell me more about how your colleagues influence how you decide what to do?
        - For what types of situations do you consult with your colleagues?
            - How do you compare the situations or cases you discuss with colleagues to the situations or cases where you don't?

  [if mention of face-to-face patient factors or goals of care?]

    - Tell me more about how these interactions with patients influences how you decide what to do?
        - How does the conversation with the patient shape your thought processes and choices between two reasonable treatment options?

  [if mention of the culture at an institution]

    - Tell me more about how the culture of your institution influences how you decide what to do?
        - Who drives this culture?

- How does this influence your individual decisions?

[if mention of guidelines/protocols/evidence/risk calculators/data, remember that they brought this up for the next section of questions]

[if mention resource availability/constrained resources]

- How does resource availability influences how you decide what to do?
  - Is this the same in all of your practice settings?

[if mention of complications/risks]

- Tell me about how [insert specifically mentioned] complications influence how you decide what to do?
- Are there certain risks/complications that are more or less important?
  - How do you weigh the value of these risks/complications with regard to overall care and treatment choice?
  - Do you quantify the likelihood of risks and benefits?
    - How do you do this quantification or estimation?

[if **no** mention of complications or risks]

- What types of potential complications or risks of treatment may have influenced your decisions when weighing options?
- Are there certain risks/complications that are more or less important?
  - How do you weigh the value of these complications with regard to overall care and treatment options?
  - Do you quantify the likelihood of risks and benefits?
    - How do you do this quantification or estimation?

- Okay, now I'd like to take a moment to talk about how you think population-level data (like from large research studies) applies, or doesn't apply, to individual patient care.

[pause]

- Tell me about what comes to your mind when I bring this up?
- How do you apply population-level data to your clinical practice?
  - How does this go well?
  - How is this difficult or limited?

- Are decision-support tools a routine part of your clinical practice?
  [if need an example, can suggest clinical practice guidelines, risk-calculators, protocols, pathways, textbook, up-to-date, etc]

  [if yes]
  - What types of decision-support tools do you use in your clinical practice?

  [if no]
  - What limits the use of decision-support tools as a routine part of your clinical practice?
  - How would they look if they were to be a beneficial part of your routine practice?

- Though population-level data can be shared or used in many ways, I want to understand specifically how risk calculators influence surgeons' decisions [if example needed:" like the NSQIP surgical risk calculator"]. Do you use them, in general? [if participant is confused about what a risk-calculator is…can say that there are many different types of risk calculators, but they all require input of some information about the patient and then give you estimates of risk of complications – they aren't guidelines, protocols, or "best practice" but try to give individualized patient information]

    [if no]
    - Are there specific patients or types of situations in which you do use them?

    [if yes, move on to **specific risk calculator example questions** below]

    [if still no]
    - Many people that I speak with don't use risk-calculators.
        - Why aren't they a part of your practice?
        - Tell me about where you think risk-calculators fall-short?
        - How would they be more useful to you in your practice?

    [if yes]
    - Has this always been the case in your practice?
    - Why did you adopt their use into your practice?
    - What are the types of situations in which you use a risk calculator?
        - Why do you use risk calculators in these cases?
        - Do you always use risk calculators in the same way?

            [if yes]
            - Tell me about how this works?
            - Tell me about why you developed this practice?

            [if no]
            - What are the different ways in which you use them?
            - What makes you choose one way or another?

        - How are cases in which you use a risk calculator different from other cases where you don't use a risk calculator?
            - Does a patient's medical history influence the likelihood of you using a risk calculator?
                - How so?

[**Specific risk calculator example questions**]

- Tell me about a specific time when you used a surgical risk calculator for one of your consults or patients.
    - Walk me through the situation
    - When in the timeline of the patient's care did you use the risk calculator?
    - What types of things were important to include in calculating the patient's risks?
    - What types of things do you remember from the output?
    - How did you think through the provided output from the risk calculator?
        - Was the output surprising to you or as you expected?
        - What was the most important information that you were looking for?
        - Did the output change any of your decisions or plans?

            [if yes]
            - How so?

    - What did it change?

    [if no]

    - Is there anything that would have changed your decisions or plans?

- Did you communicate any of the risk calculator results to the patient?

    [if no]

    - Tell me why not?
    - Tell me about what types of things you discussed with the patient?

    [if yes]

    - What aspects of the output did you communicate to the patient?
    - How did you choose what to include or what not to include in this conversation?
    - How did the patient receive this information?

eSection 3: Data Analysis

eTable1: Codebook

Code Name	Definition	*Examples*
**Physician Perception of the Patient**	Instances in which the physician refers to the patient as an influence on their thought processes/actions/steps, decisions, or predictions (don't code to parent code)	
Patient Risk	Instances in which the physician refers to the factors that contribute to the patient's risk (of needing treatment, that which is associated with treatment: surgery, non-operative management, palliative care, etc) and/or the risks themselves, including history of present illness (severity of illness, vital signs/hemodynamics, current medications, diagnostics), patient factors (age, change from baseline, comorbidities), risks/complications, prognosis.	- *"I would say that the comorbid patient is definitely the patient where we need to spend the time and understand, you know, what are the actual chances physiology making it through this?"*<br>- *"But I will say that this operation, while not without risk, is not one of the riskiest surgeries and will give us a definite answer about what's going on.*<br>- *"OK, she's not 85 years old where there's not much productive life left. She was like, I think in her 60s. So, she is technically an OK surgical candidate."*
Patient goals of care/treatment preferences	Instances in which the physician refers to the patient's goals of care and treatment preferences, including their information desires/health literacy.	- *"But that might not be the success of surgery to them, might not be an acceptable enough, good enough outcome for a procedure"*<br>- *"[establish] their expectations from surgery, and if they understand what recovery looks like, post-surgical recovery, the best possible outcome"*<br>- *"When the patient clearly didn't want to be intubated, didn't, you know, didn't want to be resuscitated, knew they were facing a terminal illness and didn't want surgery - that's a pretty easy one."*
**Environmental Influences**	Instances in which surgeons mention environmental influences that direct their thought processes/actions/steps, decisions, or predictions (don't code to parent node)	
Clinical Environment	Instances in which the surgeon discusses elements of the clinical environment as influencing/being a part of their thought processes/actions, including available resources (built, time, money, etc) and incentives.	- *"Uh, I wanna say cost, to some degree, although I don't want to waste resources on a patient who is just not gonna live, right? So, we don't say that to the patients, right? But at the end of the day, like, we do have to keep the lights on."*<br>- *"You know, I do think when you're around a situation where you're quite incentivized to operate, you see a lot of people operating, lever hungry and like to operate, and that plays into everything. I think the total stereotype is not true, but I think you have the stereotype on a place where people are quite RVU-based, is that they're just gonna operate on everything that moves."*<br>- *"I don't really think about the time of day or, you know, what else I have going on. I think, you know, I just need to think about the patient, so I try to alleviate anything else that just is clouding the picture, or whether it's your schedule, or your day, or, you know, if you're tired or not - I try not to think of those things."*
Colleagues	Instances in which the surgeon discusses their colleagues as	- *"That's where the senior-level advice comes in. So, more often than not, I've rarely been ever talked into*

	influencing/being a part of their thought processes/actions or a source of information (or when colleagues' opinions/thoughts do not impact thought processes, including disagreement) and their perception of other providers/residents doing the work of interacting with patients	*operating, it's more being talked out of operating whenever I've reached out to someone senior."*<br>- *"And I'm just getting a second set of eyes on the problem to make sure that, you know, there isn't an angle that I'm not thinking of. So, it's more, it's more of a, you know, confirmation, if you may, that yep, I'm going to do this and I just want to make sure that there is nothing else that you're thinking of."*
Culture	Instances in which surgeon discusses culture, including patient culture (self, community, national), hospital culture, or their own personal culture.	- *"Life, and end of life, is seen as inevitable and almost like a fatalistic approach that, you know, God's will plays a big role in where you are and where you're headed. So, when you are elderly, there does come a point where people and families, more so, are more, uh, amenable to understanding that yeah, these ar the limits of, you know, our time on Earth, or you know, what God's will might have to play."*<br>- *"Yeah, I think, umm, it's interesting. I do think that on the bias, compared to the two places I've worked as faculty, I would say [out hospital] is more operative than [another institution], and there were people who were, you know, on the margin of decision making, who I think if they were transported magically to another institution, either would or would not have gotten an operation"*
Population-Level Data or Book Knowledge	Instances in which surgeons discuss specific population-level data or book knowledge as driving thought processes or actions, and/or limitations of such knowledge.	- *"And the book said one thing: the book said if you see a clear injury, then you're supposed to repair it, but if you don't then – but what does that mean?"*<br>- *"And I will say that, you know, this is generalized for all-comers, obviously some people have high risk and some have low risk."*<br>- *I think, other trials that have not, kind of, not seen themselves, yet, translated into clinical relevance would be, like, not using antibiotics for, like, uncomplicated diverticulitis. Good data to do it, but emergency room providers don't tend to do that because we want to, kind of, do something or there's maybe a, sort of, [protect yourself from litigation[aspect to it, or feels bad to be like, "You have diverticulitis, go home'"*
**Physician perception of themself**	Instances in which the physician refers to themselves as influencing/being a part of their thought processes/actions, decisions, or predictions (don't code to parent code)	
Their own abilities, knowledge, or experience	Instances in which the physician refers to their own abilities, knowledge, and experiences, including limitations.	- "I was taught early on that there are […] bold surgeons and old surgeons, but no old, bold surgeons, because they have been humbled by experience."<br>- "And obviously then personal experience. I think we are humans – obviously we are humans – but I think that recent experience from another patient that has done poorly plays a big role in how aggressive or how conservative, even if that means actually you're operating, do we need to be."
How their recent experiences or	Instances in which a surgeon discussed a recent experience/pivotal case/another patient's outcomes (eg	- "So, the next two or three spleens that I do, if I'm borderline that if the pancreas is maybe OK but I'm not quite sure – if I hadn't had that complication, I

pivotal cases influence their actions	discussion of how long it changes practice and the type of change they are making, or the recognition that it should or shouldn't change their practice)	would not be leaving drains, but because of that one bad complication, the next two or three splenectomies where the pancreas was kind of borderline automatically get drained for me for 48 hours, and, again, it shouldn't change the dictum, and it shouldn't change my practice, because that one patient did poorly, probably for some other reason, not because I didn't leave a drain behind to collect something, but – because there are subclinical leaks that we never see, right? "<br>- *"I had a partner who would like change staplers, like, every time anything happened, anytime they had a leak, anytime they had a bleed, anytime anything happened, he would, he would do that."*
Their responsibility	Instances in which the physician refers to their responsibilities, including the idea of wanting to "do the right thing", their own opinions of a good/reasonable outcome, and recognition of their own limitation.	- "And you want the patients to be, you know, making the right decision, and also for the right reason. "<br>- "And, you know, is that - does that put you in a little bit of an uncomfortable position as a surgeon? Yeah, it's does, right? That feels - I was having some anxiety surrounding that, I do believe that. But part of me thinks well, gosh, like, what if we did the colectomy and maybe that would have stopped some sort of driver of their pulmonary deterioration."<br>- "If I'm offering someone an operation and I think the outcome of that is going to be a total colectomy with end ileostomy, to me, that is better than death. So I will offer that."<br>- "I think humility and being humble in the expectations of what you can achieve – because you're not working with adynamic processes, you're not working with like copper pipes and electrical wires. You're working with human beings."<br>- "To be honest with you, surgeons are disincentivized to have this conversation even when it's the right thing, right? It will take me longer to talk the family through comfort care than it would to take the colon out and just drop him off in the unit."
The way in which they deliver information (to patient or surrogate decision makers)	Instances in which the physician refers to the ways in which they deliver information, or communicate, with patients/surrogate decision makers, including communication tools, noted difficulties in communication, the idea of swaying/leading patients, etc.	- *"That is, like, where most of the really heart wrenching conversations come, and so I think that I probably, a bit depending on whether I think we need to operate or we don't, couch the, like, morbidity and, sort of, emotional/social impacts of the colostomy differently to the patient. And that's just true. And I know I do that, I don't present it objectively to all people."*<br>- *"And you have to be polite and say, "listen, I'm not God, and I don't have all the right answers, and I don't know that I'm right. But in my judgment, this is the right thing to do.""*<br>- *"I sort of do, but I'm very careful with that because again, you know, going back to that human nature thing - if I said to a family member that this patient has a 5% chance of living, 95% chance of mortality, I will tell you that the majority of people will take that 5%. And if you offer an operation, or you offer them whatever, on 5%, they're gonna take it."*

**Physician Perception of Risk Calculators**	Instances in which the physician specifically discusses risk calculators as an element of patient care and a factor that influences their thought processes/actions, decisions, and communication.	
Logistics of Use	Discussion of their current use of risk calculators (eg discussion of what information goes into the risk calculators, what is pulled from them, what patients they are used for, when/where they are used, etc)	- *"When I had more clinic time, when I was seeing patients in clinic and signing them up, scheduling them, I would absolutely use it. "*<br>- *"I would say that I would do it as part of my consent process, more so than counseling process."*<br>- *"Liver disease. Liver disease and lung disease, when they really need to formalize that and get a real sense of where they're at […] Am I gonna make this, like, dramatically worse? OK, well, let's, you know, let's get some numbers."*
Benefits	Discussion of the benefits of risk calculators (to inform/prepare patients, to consent, to recognize knowledge gaps, etc)	- *"It does inform their decision, and they're going in with eyes wide open."*<br>- *"And, in a certain way, I think in an obtuse way, it also, sort of, not only like increases the satisfaction with you, just by being up front, I think it reduces the risk of them coming back and being unhappy and pursuing some, you know, medical-legal action against a failed surgery, if you may."*
Limitations	Discussion of the limitations of risk calculators (doesn't apply to specific patients, surgeon preferences being more powerful, difficulty to know when to change practice, logistics, lack of information, etc) and the value of different types of data.	- *"I think it's the internet. It's just time. If you're running from clinic to the OR, back to clinic, to another OR, it's just time […] at that point, it's harder to go back, to bring your computer, like logistically, hen bring it up and then go through all of that again. So it's more logistics and time as opposed to belief in that tool as a beneficial informant or not."*<br>- *"Most people had pretty much made up their mind to proceed and it was putting a number to the high risk, or whatever scary things I was telling them, and put a number to that, sort of, word, but it didn't like defer anyone or change anyone's mind from proceeding so far, in my experience."*<br>- *"The just the size hasn't been put into it yet, there isn't. You know, there's probably too much of a point spread, uh or whatever, when you're trying to look at the accuracy of that just yet. There's some things that are out there, but, you know, it just - I don't know, at least for me, as far as I know, it's not quite ready for prime time."*
Future State	Discussion of the future state of risk calculators, including desires for optimal use.	- *"So, I think the future is there is gonna be more, maybe just to jog your thoughts, not to completely control your activities. But I think that, you know, we're heading down that route with all big data coming. So, I think it'll be neat."*<br>- *"I think it needs to be visible. I think it needs to incorporate a lot of data and data points, so having that live in the EMR, where it's already pulling all the data and I don't have to separately enter it, and then feel like I'm only entering half of the relevant things."*

References

1. Tong A, Sainsbury P, Craig J. Consolidated criteria for reporting qualitative research (COREQ): A 32-item checklist for interviews and focus groups. Int J Qual Health Care. 2007;19(6):349–357. Accessed Apr 3, 2023. doi: 10.1093/intqhc/mzm042.
